# Unpopularity of Private Nursing with Hospital Staffs

**Published:** 1919-11-22

**Authors:** 


### UNPOPULARITY OF PRIVATE NURSING WITH HOSPITAL STAFFS.

By F. A. G.

### II. The Ideal Home for the Private Nurse.

In order to make private nursing attractive, the question of building a separate home for the private nursing staff on the same lines as the modern nurses' home is all important. The scheme might be developed to provide that accommodation for district and visiting nurses, thus further extending the usefulness of the hospital. When once the building is established, the income derived from a sufficiently large staff will make it entirely self-supporting, but the nurses should be given adequate remuneration.

All classes of the community are served by providing district and visiting nurses, and the nurses should take these duties, in addition to private work, in turn. Therefore a sufficiently high fee to meet all expenses should be charged to the wealthy patient for the services of a well-trained nurse. But consideration could always be given to necessitous cases. The building should be not merely an institution from whence nurses may be obtained, but a home in the truest sense of the word to which a nurse may look forward to going when her case is ended. It should be built on exactly the same lines as the modern nurses' home, and be sufficiently large to give each member of the staff her own bedroom, cosily furnished, within which she may create her own atmosphere. Then, when she goes out to a new case, her possessions may be collected and covered with dust-sheets, and the room locked, just as a sister's room is when she goes for her holiday. In many instances patients would be only too thankful she should return to her home to sleep, taking the day or night as the case might be. When she is expected in from a case, the room would be cleaned and made ready for her, and a few fresh flowers would make it "home." A spare room should always be kept in readiness for the nurse who may come in unexpectedly until her own can be got ready for her.

In addition to a bedroom for each nurse on the staff, there should be a general sitting-room, a recreation room, visitors' or waiting-room, and silence room. There should also be a pantry, wherein she may make the cup of tea she so dearly loves, and do little bits of washing and ironing. The sister-in-charge would have her own quarters, and she would conduct the business relative to the staff. These rooms, together with a liberal number of bathrooms, box- and linen-rooms, lavatories, and a lift, would complete the home.

In order to ensure that the private nurse be kept in touch with hospital life, and that she should have every opportunity of keeping her knowledge up to date, she should go on duty in the wards or various departments of the hospital when in from a case, after sufficient rest. She ought not to be isolated altogether from the hospital staff, and the principal meals of the day should be taken in the hospital dining-room, if space allows, at a table reserved for the private staff. In institutions where it is desired to keep a strict account of the expenses of the staff, a kitchen and dining-room might have to be built in the home. Tea, in any case, should be served in their own sitting-room, and breakfast in bed, until sufficient rest has been obtained. The domestic work in the home would naturally fluctuate, and the number of servants required might be a difficult problem, but this would be overcome by occasional daily help.

The private nurse undoubtedly needs a home when in from a case, as her hours of duty are frequently long and her work arduous. It is impossible for her to conform to set rules and hours with regard to her working day, especially when her patient is in the acute stage of illness; and no nurse who has the true vocation would expect to do so. Often during convalescence a fair amount of relaxation is possible, but hospitals can do much to help their nurses secure their rights by sending out printed instructions to relatives with the case papers, stating the minimum amount of off-duty time which should be allowed, and a note to the effect that a nurse is only responsible for duties connected with her patient, would help her in some families where she

The Previous article appeared Nov 8, p ix.

might otherwise be expected to undertake the duties of a general servant in addition to those of a nurse. This would not prevent the nurse from using her discrimination as to what help she may give in a household under certain circumstances. Lastly arises the question of uniform and title, and I would suggest that the private nurse should wear a distinctive uniform and be addressed as sister, so as to avoid confusion with the children's nurse in houses where one is kept.

The title of "Sister" would be easily adopted if the word were substituted for "Nurse" on the case papers, and it would also give the nurse a higher social standing. The scheme may be termed idealistic, but does not the constant aim to attain ideals add savour to life? In the hands of the right woman such an establishment as we have sketched could become a real home. There would be no dearth of really good nurses to fill the ranks of the private staff.

